# CircSMYD4 regulates proliferation, migration and apoptosis of hepatocellular carcinoma cells by sponging miR-584-5p

**DOI:** 10.1186/s12935-020-01648-3

**Published:** 2020-11-19

**Authors:** Yanhe Zhang, Hui Wang, Chao Li, Linlin Gao, Yayun Zheng, Wenjuan Chang, Chao Lu, Xiaoguang Zhao

**Affiliations:** 1 Department of Gastroenterology, Jiaozuo People’s Hospital, Jiaozuo, China; 2 Department of Medical Oncology, Jiaozuo People’s Hospital, No. 267, Middle Jiefang Road, Shanyang District, Jiaozuo, 454002 China

**Keywords:** Hepatocellular carcinoma, CircSMYD4, miR-584-5p, Circular RNA, Proliferation

## Abstract

**Background:**

There is evidence that circSMYD4 is differentially expressed in hepatocellular carcinoma (HCC), but its mechanism of action remains unclear. Therefore, this study aimed to explore the role of circSMYD4 in the occurrence and development of HCC and its specific molecular mechanism.

**Methods:**

The expressions of related genes and proteins in the development of HCC were detected by real-time quantitative-PCR and Western blot. HCC cells treated with RNase R and Actinomycin D were used to examine the stability of circSMYD4. Bioinformatics analysis, RNA pull-down assay, luciferase assay and *Spearman* correlation analysis were performed to evaluate the interaction between circSMYD4 and miRNA. Cell Counting Kit-8, clone formation assay, wound healing assay, Transwell, flow cytometry, nude tumor formation experiment, and immunohistochemistry were employed to analyze the function of circSMYD4 in HCC. A rescue experiment was conducted to analyze the effect of miR-584-5p on the physiological functions of cells.

**Results:**

CircSMYD4 was down-regulated in HCC tissues and cells, and was not easily affected by RNase R and Actinomycin D. The abundances of circSMYD4 and SMYD4 in the cytoplasm were significantly higher than in the nucleus. Up-regulation of circSMYD4 inhibited the proliferation, invasion and migration and promoted the apoptosis of HCC cells in vitro, while it inhibited tumor growth, promoted apoptosis-related proteins, and suppressed alpha-fetoprotein (AFP) levels in vivo. CircSMYD4 could be used as a miRNA sponge to target miR-584-5p. In addition, miR-584-5p overexpression partially reversed the regulatory effect of circSMYD4 on HCC.

**Conclusion:**

CircSMYD4 prevents the development of HCC through regulating multiple signaling pathways such as metastasis and apoptosis by sponging miR-584-5p.

## Introduction

Liver cancers are the fourth most common cause of cancer-related deaths worldwide, and it ranks sixth in incidence cases. According to annual forecasts, the World Health Organization estimates that more than 1 million patients will die of liver cancer in 2030 [1]. Hepatocellular carcinoma (HCC), which typically develops on a background of chronic liver disease, is the most important type of primary liver cancer [2]. The development of HCC is a complex multistep process involving persistent inflammatory damage, including hepatocyte necrosis and regeneration associated with fibrotic deposition [2]. In recent years, significant development has been made in the treatment methods for HCC, including liver cancer resection, liver transplantation, radiofrequency ablation, interventional therapy and targeted drug comprehensive treatment, but the metastasis and recurrence rates of HCC after surgery are still high [3–5]. Therefore, studying the etiology and exact molecular mechanism of HCC and to identify new targets and prognostic predictors through molecular profiling is helpful to develop new treatment strategies for HCC.

As an important component of non-coding RNA, circular RNA (circRNA) has attracted more and more attention from scholars due to its unique structure and potential functions [6]. Unlike linear RNA molecules, circular RNA is a class of covalently closed circular RNA molecules without a 5′ end cap and a 3′ end polyadenylation tail [7]. Recent studies have found that circRNAs play a necessary role in regulating RNA networks, considering the competitive RNA–RNA interactions and the high complementarity of circRNA to its linear mRNA [8]. Many circRNAs have been proved to be highly expressed in tissue-specific or cell-type-specific ways, and are regulated in the process of human epithelial–mesenchymal transformation, which suggests that the differentially expressed circRNAs may contribute to cancer biology [9]. Owing to the closed-loop structure, circRNA is not affected by exonuclease and other factors and has a stable expression, thus possessing the potential to become a tumor biomarker [10]. Hence, it is of great importance to study the role of circRNA in the occurrence and development of HCC and its specific molecular mechanism, and to explore whether it can serve as a stable biomarker in the early diagnosis, therapeutic intervention and prognosis analysis of HCC. The role of circRNA in the development of HCC has also been reported [11–14]. For example, circ-MTO is substantially down-regulated in HCC tissues, the survival time of patients with low expression of circ-MTO is greatly shortened, and it can adsorb miRNA-9 to inhibit important physiological functions such as HCC proliferation and invasion [15]. Some scholars have reported that circRNA cSMARCA5 is significantly down-regulated in HCC tissues, and its expression level is closely related to invasion characteristics [9]. In addition, circRNA cSMARCA5 can promote the expression of the tumor suppressor gene TIMP3 by adsorbing miR-17-3p and miR-181-5p, thereby inhibiting the growth and metastasis of HCC [9]. Although many non-coding RNAs have been found to be deregulated in HCC with potential clinical applications. However, it is still necessary to identify the expression profile, function and deregulation of circRNA in HCC.

Previous reviews have found that hsa_circ_0004018 (circSMYD4) has a significantly lower level in liver cancer tissues than in adjacent tissues and possesses supersensitivity to alpha-fetoprotein (AFP), and thus it can be used as a potential biomarker for the diagnosis of HCC [16, 17]. However, the mechanism of circSMYD4 in HCC has not been reported. Hence, we aimed to examine the role of circSMYD4 in the occurrence and development of HCC and its specific molecular mechanism, in order to provide a basis for the potential value of circSMYD4 in the diagnosis and treatment of HCC.

## Materials and methods

### Ethics statement

This study was approved by the ethics committee of Jiaozuo People’s Hospital (GA201906021). All HCC patients and their families signed informed consent. All animal studies were conducted following the laboratory guidelines for animal care and were approved by the Institutional Animal Care and Use Committee (DG201908102) at Jiaozuo People’s Hospital.

### Tissue, cell and culture

Total 40 patients who received surgical resection from July 2018 to January 2019 in Jiaozuo People’s Hospital were selected. Inclusion criteria: patients diagnosed with HCC by histopathology; exclusion criteria: patients reported no history of chemotherapy or radiation before surgery. The clinicopathological characteristics of patients list in Table 1. Cancerous tissues and adjacent tissues (ANT) (> 3 cm from the edge of the cancerous tissues) were collected from patients after surgical resection. All tissues were stored at − 80 °C after resection.

**Table 1:** Correlation between the clinicopathologic characteristics and circSMYD expression in hepatocellular carcinoma

Characteristics	CircSMYD expression		*P*
	Low (n = 20)	High (n = 20)	
Age (years)			0.744
< 50	8	7	
≥ 50	12	13	
Sex			0.490
Male	15	13	
Female	5	7	
HBV			0.736
Absent	13	14	
Absent	7	6	
Serum AFP level (ng/mL)			0.327
< 20	9	6	
≥ 20	11	14	
Tumor size (cm)			0.010
< 5	4	12	
≥ 5	16	8	
No. of tumor nodules			0.490
1	15	13	
≥ 2	5	7	
Cirrhosis			0.197
Absent	10	6	
Absent	10	14	
Venous infiltration			0.749
Absent	12	11	
Absent	8	9	
Edmondson–Steiner grading			0.027
I + II	6	13	
III + IV	14	7	
TNM tumor stage			0.004
I + II	5	14	
III + IV	15	6	

*HBV* hepatitis B virus, *AFP* alpha-fetoprotein, *TNM* tumor-node-metastasis

THLE-2 cell line (CRL-2706) and HCC cell lines (SNU-182 (CRL-2235), SNU-387 (CRL-2237), SNU-423 (CRL-2238), Hep3B (HB-8064), PLC/PRF/5 (CRL-8024), SK-Hep1 (HTB-52)) were procured from the American Type Culture Collection (ATCC, USA), and cultured in strict accordance with the requirements of the ATCC. All cells were cultured in DMEM (10566024, Gibco) containing 10% FBS (16140071, Gibco, USA) in a cell incubator at 37 °C with 5% CO_2_.

### RNase R treatment and actinomycin D

Total RNAs of circSMYD4 and SMYD4 (10 μg) were incubated with RNase-R (40U, Epicentre Technologies, USA) at 37 °C for 60 min to analyze their RNase R resistance [18]. The cell culture medium was added with Actinomycin D (2 mg/mL, 129935, Millipore, USA), and the half-lives of circSMYD4 and SMYD4 were evaluated and analyzed. After treatment with Actinomycin D or RNase R, the expression levels of circSMYD4 and SMYD4 were detected by RT-qPCR.

### Cell transfection

CircSMYD4 overexpression (circSMYD4) and interference plasmid (si-circSMYD4) were purchased from Genepharma Co., Ltd. (Shanghai, CA). Blank pcDNA3.1 (V79520, ThermoFisher, USA) vector was used as a negative control. MiR-584-5p mimic (miR10003249-1-5), siNC (siN0000001-1-5), and mimic control (miR1N0000002-1-5) were obtained from RIBOBIO Co., Ltd. (Guangzhou, CA). The circSMYD4 junction sequence is 5′-TACAACACACAGACGTGTCTTAAAG-3′. SNU-387 and SK-Hep1 cell lines (5 × 10^5^/well) were inoculated into 6-well plates, and when cultured to reach 50% confluence, the cells were subjected to transfection using Lipofectamine 3000 according to the instructions (L3000015, ThermoFisher, USA).

### Real-time quantitative PCR

For nuclear and cytoplasmic RNA extraction, a Cytoplasmic & Nuclear RNA Purification Kit (Cat. 21000, 37400, NorgenBiotek, Canada) was used. Total RNA was extracted using TRIzol reagent (15596018, Invitrogen, CA), and its concentration was measured. Then 1 μg of RNA was taken to synthesize cDNA using a reverse transcription kit (One Step PrimeScript RT-PCR Kit, RR064A, Japan) in accordance with the instructions. The cDNA was diluted to a certain concentration as a template, and RT-PCR was performed with a RT-PCR kit (A46113, Applied Biosystems, USA) following the instructions. Three duplicate wells were set up for each sample. The relative expression levels of mRNA and miRNA were quantified using the 2^−ΔΔCt^ method [19], and GAPDH and U6 were selected as internal parameters. Table 2 listed all the primers used.

**Table 2:** All primer in this study

ID	Forward sequence(5′–3′)	Reverse sequence(5′–3′)
circSMYD4	TCAACCTTTTGCCCCACACT	AAGACACGTCTGTGTGTTGT
GAPDH	TCGACAGTCAGCCGCATCTTCTTT	ACCAAATCCGTTGACTCCGACCTT
SMYD4	GGTGGGAAAGGACTCGGAC	GGTTAGCATGACACAGTGACAT
U6	AGCCCGCACTCAGAACATC	GCCACCAAGACAATCATCC
miR-660-5p	TGCATATCGGAGTTGGTCGT	GTCGTATCCAGTGCGTGTCG
miR-584-5p	ATGGTTTGCCTGGGACTGAG	GTCGTATCCAGTGCGTGTCG
miR-149	TTCACTCCCGTGCTTGTCC	GTCGTATCCAGTGCGTGTCG
miR-127-5p	GGGCTCTGATGTCGTATCCAG	GTCGTATCCAGTGCGTGTCG
miR-326	CATCTGTCTGTTGGGCTGGA	GTCGTATCCAGTGCGTGTCG
Bcl-2	GTCTTCGCTGCGGAGATCAT	CATTCCGATATACGCTGGGAC
Bax	CCCGAGAGGTCTTTTTCCGAG	CCAGCCCATGATGGTTCTGAT

### Cell counting kit-8 (CCK-8)

Twenty four hours after transfection of SNU-387 and SK-Hep1 cell lines, 100 μL of cell suspension (about 1 × 10^4^ cells/well) was inoculated into 96-well plates, and cultured in incubators for 0, 24, and 48 h. After incubation, 10 μL/well CCK-8 solution (HY-K0301, MedChemExpress, USA) was added into the cells. Then the cells were cultured at 37 °C for 2 h in the dark, and the absorbance at 450 nm was measured with a GX71 microplate reader (Olympus, Japan).

### Colony formation assay

Twenty four hours after transfection, SNU-387 or SK-Hep1 cells (1 × 10^3^ cells/well) were seeded in a 60 mm petri dish and cultured for 14 days. Cell colonies were fixed with methanol (34860, Sigma-Aldrich, Germany) for 15 min, and stained with 0.5% crystal violet (C0775, Sigma-Aldrich) at room temperature for 30 min. Then the cells were counted and photographed, and the clone formation rate was calculated.

### Wound healing assay

SNU-387 or SK-Hep1 cells (5 × 10^5^ cell/well) were seeded in 6-well plates. Wound was then created on a monolayer of cells using a sterile pipette tip. After routine culture for 48 h, photographs were taken and the cell migration rate was calculated.

### Transwell

Forty eight hours after transfection, SNU-387 or SK-Hep1 cells were digested with trypsin and then centrifuged. After centrifugation, the supernatant was discarded and the cells were resuspended in a serum-free medium. Afterwards, 50 mg/L Matrigel (354230, BD, USA) was diluted at 1:8, and then 50 μL of the diluted Matrigel was used to coat the bottom membrane of the Transwell chamber (CLS3398, Sigma, Germany). Next, 100 μL of cell suspension (1 × 10^5^ /well) was seeded into the upper chamber, and 500 μL of DMEM medium containing 10% FBS was added to the lower chamber. After 48 h, the chamber was removed, the culture medium in the upper chamber was discarded, the non-invasive cells on the top of the membrane were wiped off with a cotton swab, and the invading cells were counted.

### Flow cytometry

After culture for 48 h, the cells were digested with trypsin and the supernatant was discarded. Then the cells were resuspended in PBS to adjust the cell concentration to 1 × 10^4^ cells/well. The required solution was prepared using Annexin V/ PI (APOAF, Sigma, Germany) according to the instructions. The cell suspension was mixed with AnnexinV-FITC Binding Buffer and incubated at room temperature for 15 min, and then stained with PI in the dark for 10 min (final concentration 1 μg/ml). The apoptosis rate was analyzed by flow cytometry (FACScan, BD Biosciences, USA) using flow Jo V10 software (BD Biosciences).

### Pull-down assay

The 3′UTR biotinylated circSMYD4 probe and control probe were purchased from Genepharma (Shanghai, China). The circSMYD4 probe and the control probe were transfected into SNU-387 or SK-Hep1 cells and cultured overnight. The cell lysate was then incubated at 4 °C with streptavidin-coated magnetic beads to pull down the biotin-conjugated RNA complex. Finally, the bound miRNAs were extracted using Trizol reagent, and the data were analyzed by RT-qPCR [20].

### Bioinformatics prediction

CircInteractome (https://circinteractome.nia.nih.gov/) was used to analyze the target miRNA of CircSMYD4.

### Dual luciferase activity assay

The pmirGLO (E1330, Promega, CA, USA) was used to construct a recombinant fluorescent reporter vector. The wild-type or mutant circSMYD4 3′UTR reporter plasmid was co-transfected with miR-584-5p mimic or mimic control into SNU-387 or SK-Hep1 cells. Forty eight hours after the plasmid was transfected, 20 μL of the protein supernatant was added to 50 μL of the firefly luciferase substrate and mixed thoroughly, and the luciferase activity was measured. Then, the stop solution and Renilla luciferase substrate were used to measure Renilla luciferase activity. The luciferase detection kit was purchased from TransGen Biotech (FR201-01, CA).

### Western blot

For the western blot [21], after the cells were fully lysed, total protein was extracted using RIPA lysate (P0013, Beyotime, CA). Then the protein loading buffer was added to the protein and mixed, and then the mixture was boiled for 10 min. Next, a 15 μg protein sample was added to each well and electrophoresed (at 80 V for 40 min, at 120 V for 80 min), and susbsequently transferred to a PVDF membrane (at 210 mA for 100 min) (Immobilon-P Transfer Membrane, EMD Millipore Corporation, MA). The membrane was blocked with 8% skim milk for 2 h, and then incubated with the primary antibody overnight at 4 °C, followed by incubation with the corresponding secondary antibody for 2 h. An ECL kit (#6883, Cell Signaling Technology, USA) was used for chemiluminescence development, and ImageJ (version 5.0, Bio-Rad, USA) software was used for semi-quantitative analysis. The primary antibodies were as follows: E-Cadherin (1:10,000, 97kD, ab40772, Abcam, UK), N-Cadherin (1 µg/ml, 130kD, ab18203), Vimentin (1:1000, 54kD, ab92547), GAPDH (1:500, ab8245, 36kD), cleaved Caspase-3 (1 µg/ml, 17kD, ab2302), Bcl-2 (1:500, 26kD, ab59348), Bax (1:1000, 21kD, ab32503); the secondary antibodies were Goat Anti-Mouse IgG H&L (1:5000, ab205719) and Goat Anti-Rabbit IgG H&L (1:5000, ab205718); and the markers were PR1910 (11-180kD) and PR1920 (11-245kD) (Solarbio, CA).

### Tumor formation assay

SNU-387 or SK-Hep1 cells were suspended in a serum-free medium, and the cell concentration was adjusted to 1 × 10^6^ cells/mL. BALB/c nude mice (5 weeks old, male) were purchased from Beijing Vital River Laboratory Animal Technology Co., Ltd., Beijing, China. The mice were injected subcutaneously with the same number of SNU-387 or SK-Hep1 cells (n = 8 in each group) to establish a xenograft nude mouse model. A vernier caliper was used to measure the longest diameter (L) and the shortest diameter (W) of the transplanted tumor every 3 days. After about 10 days, when the tumor volume reached about 100 mm^3^, circSMYD4 and agomir-miR-584-5p, alone or in combination (10 nM), were injected into the tumor every two days. After the experiment (35 days), the mice were sacrificed by injection of excessive sodium pentobarbital (intraperitoneal, B5646-50 mg, ApexBio, USA). Then the tumor was completely dissected and photographed, and the tumor volume and weight were measured. Tumor tissue was then collected for further experiments.

### Immunohistochemistry (IHC)

After depariffinization and hydration, the tissue sections were repaired using the method of citrate buffered high-temperature and high-pressure antigen retrieval. After the sections were washed three times with PBS, the primary antibody AFP (1:100, ab46799, Abcam, UK) was added into the sections and incubated for 30 min, and then the corresponding secondary antibody [Goat Anti-Rabbit IgG H&L (HRP) (ab205718)] was added to incubate the sections for 30 min. Then the sections were immersed in a methanol solution containing 10% hydrogen peroxide for 10 min, developed with DAB for 3 min, washed with distilled water for 10 min, counterstained with hematoxylin for 3 min, and washed with distilled water for 10 min. After dehydration and transparentization, the sections were sealed with neutral resin [20]. Immunostaining images were analyzed with a microscope (Olympus FluoView FV1000, Tokyo, Japan).

### Statistical analysis

The data were presented as mean ± SD. Statistical analyses were performed using SPSS 21.0 statistical software (Chicago, IL, USA). Differences were compared using Student’s *t* test or one-way analysis of variance. The correlation between miR-584-5p and circSMYD4 expressions was analyzed using Spearman correlation coefficients. The *χ*^2^ test was carried out to explore the correlation between circSMYD4 levels and clinical features. *p* < 0.05 was defined as statistically significant.

## Results

### CircSMYD4 with a stable ring structure was down-regulated in HCC tissues and cells

**Fig. 1:**
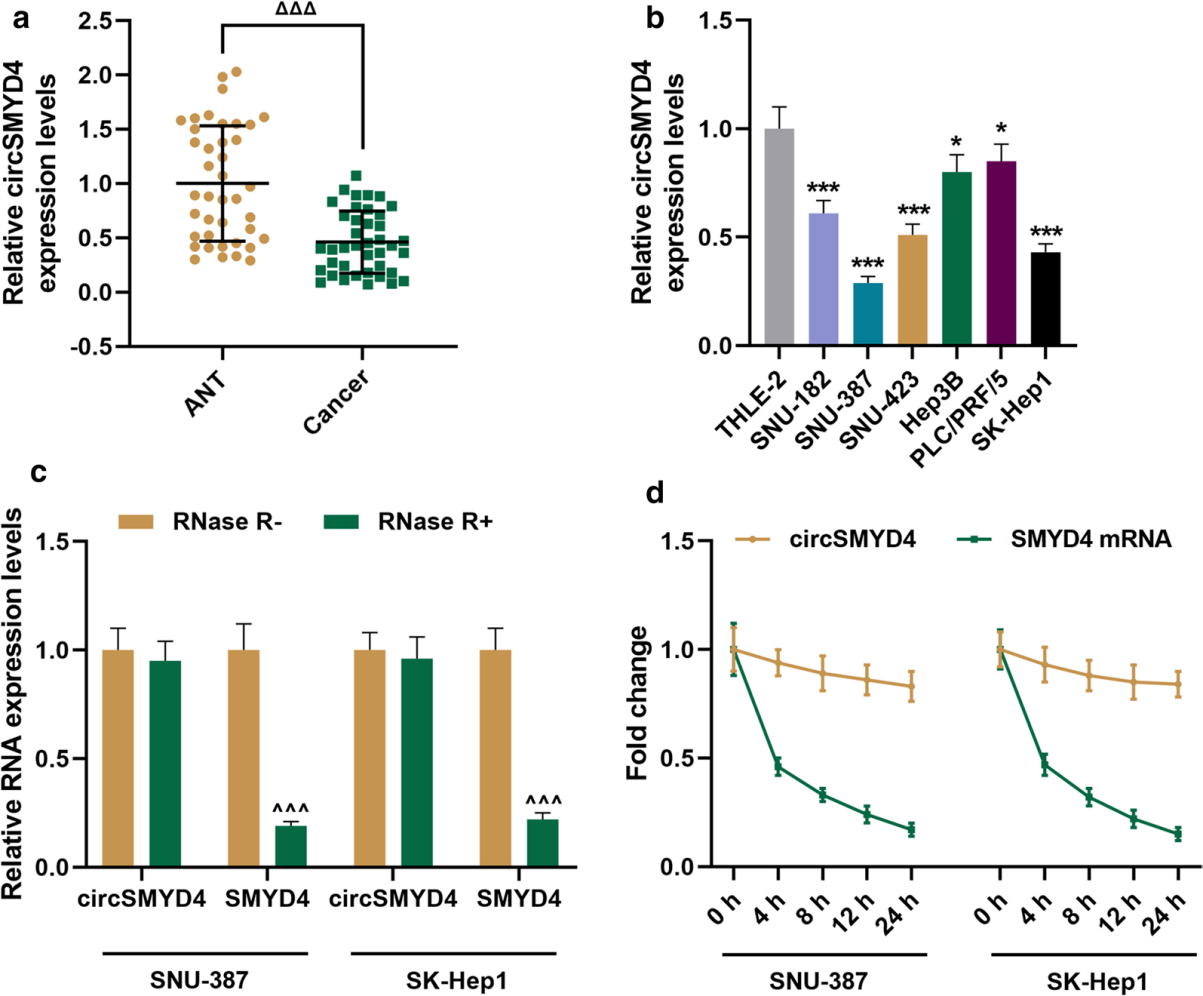
CircSMYD4 with a stable ring structure was down-regulated in HCC tissues and cells. **a** The expression of circSMYD4 in liver cancer tissues and adjacent tissues (ANT) from 40 patients was detected by RT-qPCR. **b** The expression of circSMYD4 in normal cell lines and different HCC cell lines was detected by RT-qPCR. **c** After SNU-387 and SK-Hep1 cells were treated with RNase R, the stabilities of circSMYD4 and SMYD4 mRNA were analyzed by RT-qPCR. **d** Abundances of circSMYD4 mRNA in Actinomycin D-treated SNU-387 and SK-Hep1 cells at specified time points were detected by RT-qPCR. Both biological and technical replicates were examined three times. GAPDH was used as a control. The experiment was repeated for 3 times. ^ΔΔΔ^*p* < 0.001 vs. ANT; ^*^p < 0.05, ^***^p < 0.001 vs. THLE-2; ^*^^^*^*p* < 0.001 vs. RNase R-. *HCC* hepatocellular carcinoma, *RT-qPCR* reverse transcription real time quantitative polymerase chain reaction

In order to determine the role of circSMYD4 in HCC, we first detected the expression of circSMYD4 in HCC tissues and ANT from 40 patients, and found that circSMYD4 was downregulated in HCC tissues (*p* < 0.001, Fig. 1a). We used the median of the cohort as a cut-off value, HCC patients were divided into two low/high circSMYD4 groups. As shown in Table 1, circSMYD4 expression was negatively correlated with large tumor (*p* = 0.010), high edmondson-steiner grading (*p* = 0.027) and TNM tumor stage (*p* = 0.004). We then investigated whether circSMYD4 had the same expression pattern in HCC cells, and the results demonstrated that the expression of circSMYD4 was memorably lower in HCC cell lines (SNU-182, SNU-387, SNU-423, Hep3B, PLC/PRF/5, and SK-Hep1) than in normal liver cells (THLE-2) (*p* < 0.05, Fig. 1b). Moreover, the expression of circSMYD4 decreased most significantly in SNU-387 and SK-Hep1 cells (*p* < 0.001, Fig. 1b) Later, we analyzed the stability of circSMYD4, and observed that after RNase R treatment, SMYD4 levels decreased significantly, while circSMYD4 levels remained unchanged (*p* < 0.001, Fig. 1c). Furthermore, after treatment with the transcription inhibitor Actinomycin D, the half-life of circSMYD4 exceeded 24 h, while that of linear SMYD4 was only about 4 h, which further illustrated that circSMYD4 was highly stable (Fig. 1d).

### Effects of circSMYD4 on the proliferation, migration, invasion and apoptosis of HCC cells

**Fig. 2:**
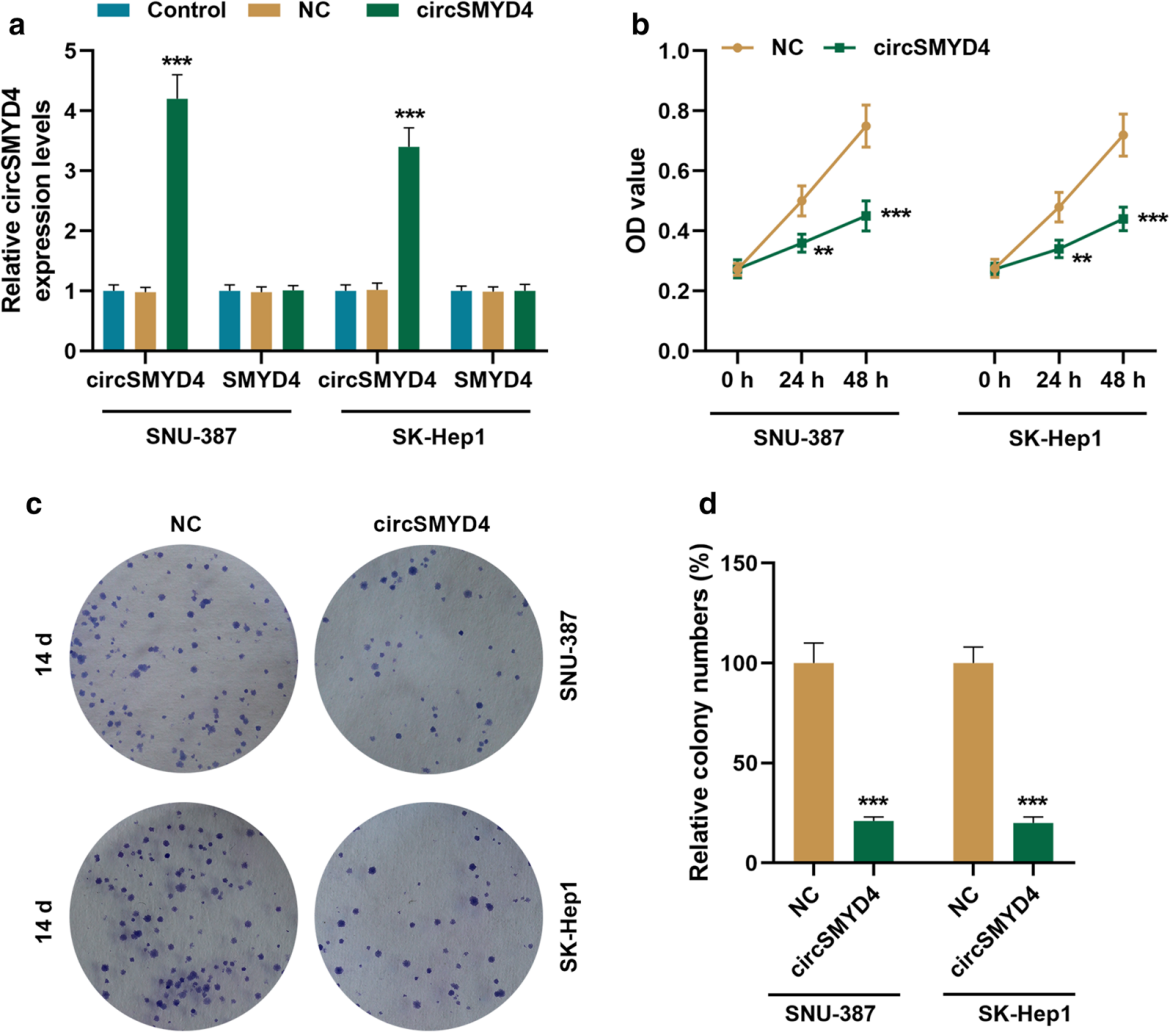
Effect of circSMYD4 on the viability and proliferation of HCC cells. **a** RT-qPCR was performed to determine the transfection rate of circSMYD4. **b** The effect of circSMYD4 on the activity of SNU-387 or SK-Hep1 cells was analyzed by CCK-8. **c**, **d** The effect of circSMYD4 on the proliferation of SNU-387 or SK-Hep1 cells was examined using clone formation assay. Both biological and technical replicates were examined three times. GAPDH was used as a control. The experiment was repeated for 3 times. ^**^p < 0.01, ^***^p < 0.001 vs. NC. *HCC* hepatocellular carcinoma

**Fig. 3:**
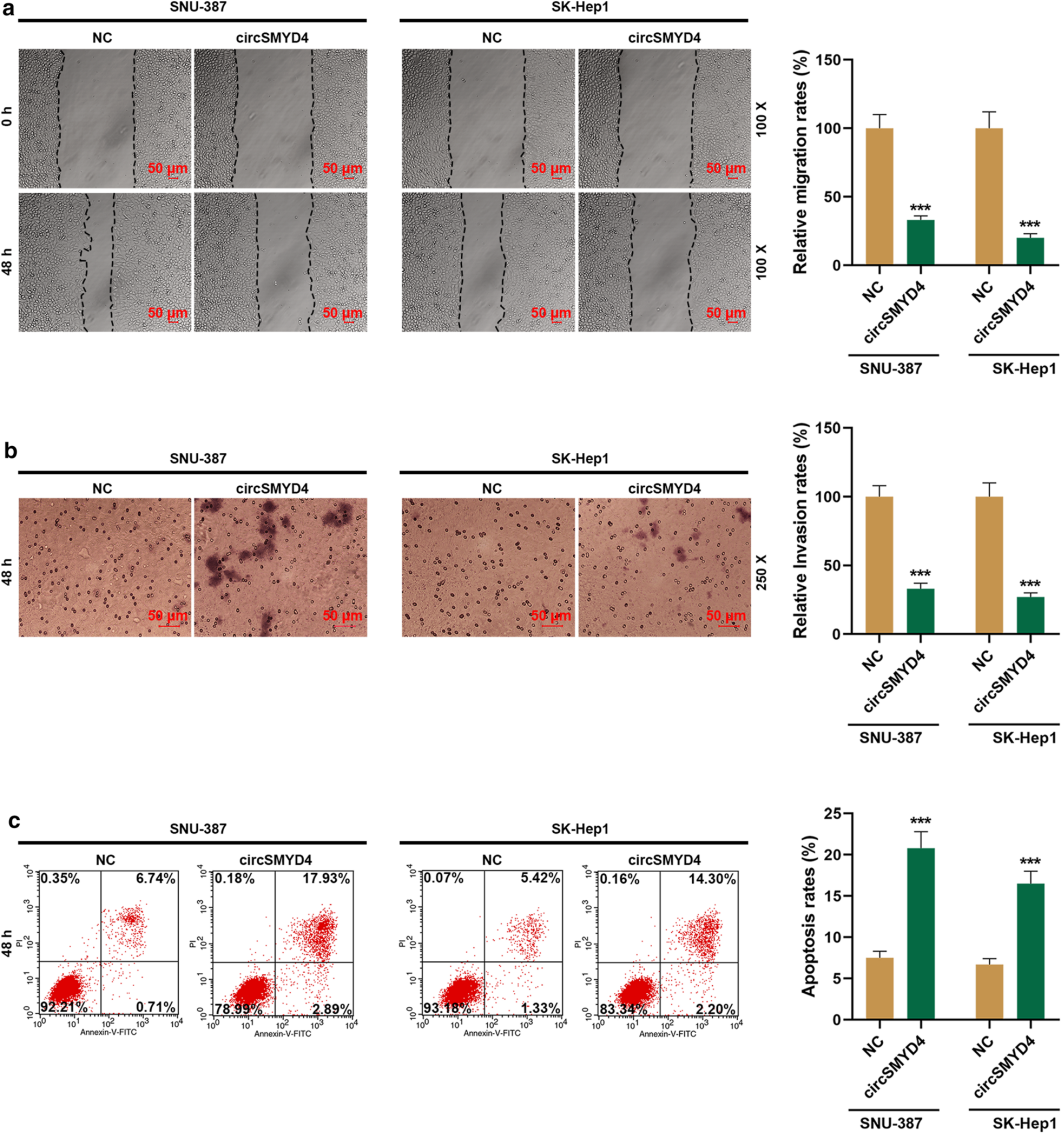
Effects of circSMYD4 on the migration, invasion and apoptosis of HCC cells. **a** The effect of circSMYD4 on the migration ability of SNU-387 or SK-Hep1 cells was detected by wound healing assay. Magnification × 100, Scale = 50 μm. **b** The effect of circSMYD4 on the invasion ability of SNU-387 or SK-Hep1 cells was detected by Transwell. Magnification × 250, Scale = 50 μm. **c** The effects of circSMYD4 on the apoptosis of SNU-387 or SK-Hep1 cells were detected by flow cytometry. Left upper quadrant represents cell fragments on the behave of FITC positive; Left lower quadrant represents the live cells because of FITC negative and PI negative; and the right upper quadrant represents late apoptotic cells on behalf of PI positive; And early apoptotic cells was designed as FITC positive and PI negative and located in the right lower quadrant. The experiment was repeated for 3 times. ^***^p < 0.001 vs. NC. *HCC* hepatocellular carcinoma

The expression of circSMYD4 in SNU-387 and SK-Hep1 cells increased significantly after SNU-387 and SK-Hep1 cells transfected with circSMYD4 overexpression plasmid, while the expression of its host gene SMYD4 was not affected (*p* < 0.001, Fig. 2a). Overexpression of circHIPK3 significantly inhibited the activity and clone formation of SNU-387 and SK-Hep1 cells (*p* < 0.01, Fig. 2b–d). Compared with the NC group, the migration and invasion rates of HCC cells in the circSMYD4 group were significantly reduced (*p* < 0.001, Fig. 3a, b). In addition, as shown in Fig. 3c, the apoptosis rate of the circSMYD4 group was markedly higher than that of the NC group (*p* < 0.001, Fig. 3c).

### CircSMYD4 as a miRNA sponge could target miR-584-5p

**Fig. 4:**
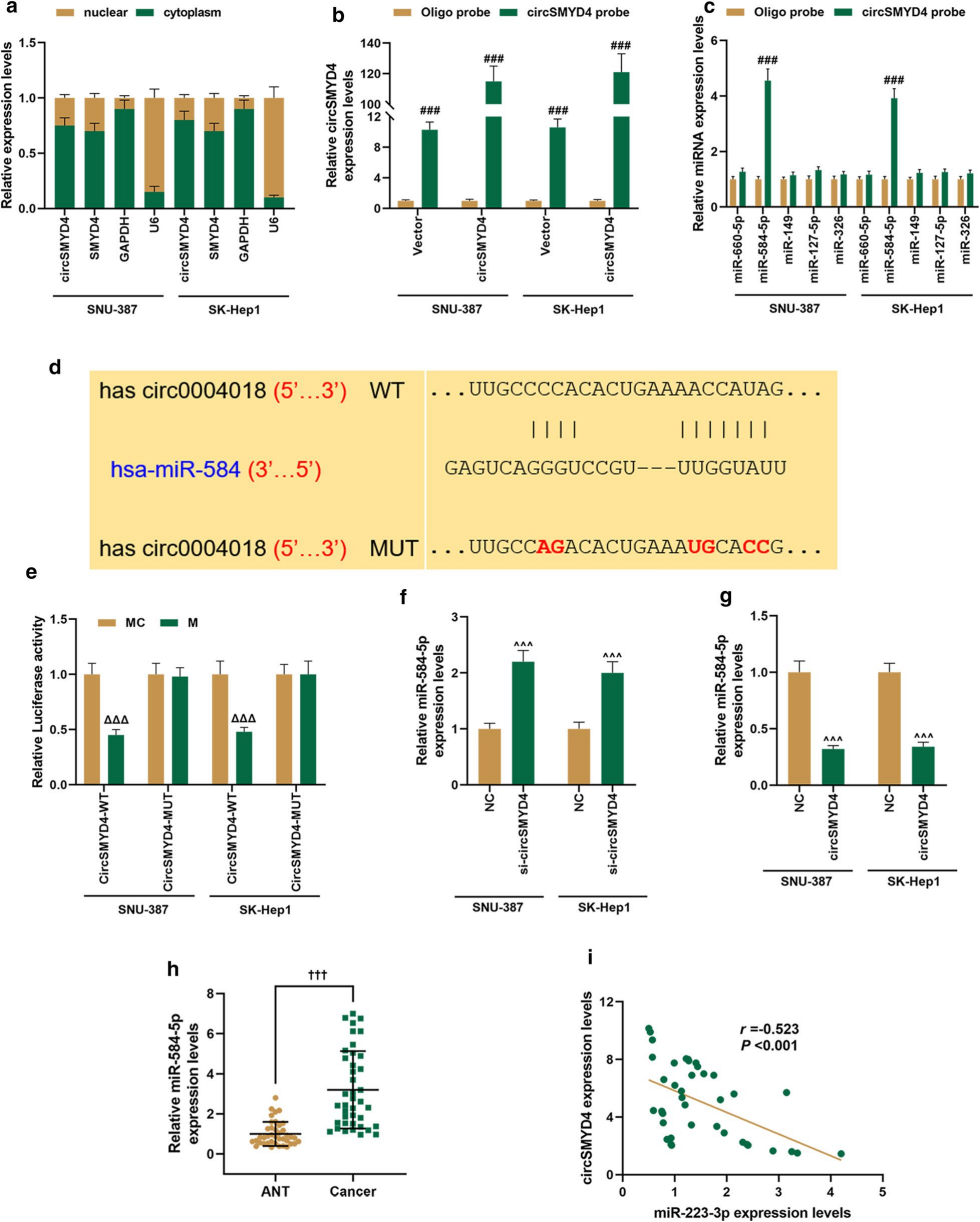
CircSMYD4 as a miRNA sponge could target miR-584-5p. **a** Cytoplasmic and nuclear RNA was isolated from SNU-387 or SK-Hep1 cells. The relative expression level of circSMYD4 in the cytoplasm or nucleus was detected by RT-qPCR. GAPDH was used as the cytoplasmic control and U6 was used as the nuclear control. **b** CircSMYD4 in the SNU-387 or SK-Hep1 lysate was extracted and enriched with a 3′ UTR biotinylated circSMYD4 specific probe, and then detected by RT-qPCR. **c** The relative levels of five candidate miRNAs in SNU-387 or SK-Hep1 lysates were detected by RT-qPCR. **d** CircInteractome and **e** dual luciferase reporter gene assay were employed to predict and validate the binding site for circSMYD4 on miR-584-5p, respectively. **f**, **g** The effect of silenced or overexpressed circSMYD4 on miR-584-5p expression was detected by RT-qPCR. **h** The expression of miR-584-5p in adjacent tissues (ANT) and liver cancer tissues was analyzed by RT-qPCR. **i** The correlation between miR-584-5p and circSMYD4 expression was analyzed using Spearman correlation coefficients. The experiment was repeated for 3 times. ^***^p < 0.001 vs. nuclear; ^*###*^*p* < 0.001 vs. Oligo probe; ^ΔΔΔ^*p* < 0.001 vs. MC; ^*^^^*^*p* < 0.001 vs. NC; ^†††^*p* < 0.001 vs. ANT. RT-qPCR: reverse transcription real time quantitative polymerase chain reaction

The abundances of circSMYD4, SMYD4, and GAPDH in the cytoplasm were significantly higher than in the nucleus. U6 was mainly located in the nuclei of SNU-387 and SK-Hep1 cells (Fig. 4a). We then used CircInteractome to predict the possible target miRNAs of circSMYD4, and found five candidate target miRNAs (miR-660-5p; miR-584-5p; miR-149; miR-127-5p; miR-326). Next, it was found that miR-584-5p was most substantially expressed in both SNU-387 and SK-Hep1 cells (*p* < 0.001, Fig. 4b, c). As shown in Fig. 4d, CircInteractome predicted the target binding site for circSMYD4 on miR-584-5p. Moreover, in luciferase reporter, cells co-transfected with mutant circSMYD4 or wild-type circSMYD4 and miR-584-5p mimic, there was no significant change of luciferase activity in wild-type circSMYD4, but a significant reduction of luciferase activity in wild-type circSMYD4(*p* < 0.001, Fig. 4e). Next, RT-PCR results showed that si-circSMYD4 up-regulated miR-584-5p and circSMYD4 down-regulated miR-584-5p (*p* < 0.001, Fig. 4f, g). We further verified that the expression of miR-584-5p in HCC tissues was substantially higher than the ANT group (*p* < 0.001, Fig. 4h). Spearman correlation analysis showed that miR-584-5p was negatively correlated with circSMYD4 expression (*r* = − 0.523, *p* < 0.001, Fig. 4i).

### Overexpression of miR-584-5p partially reversed the effects of circSMYD4 on cell proliferation and metastasis

**Fig. 5:**
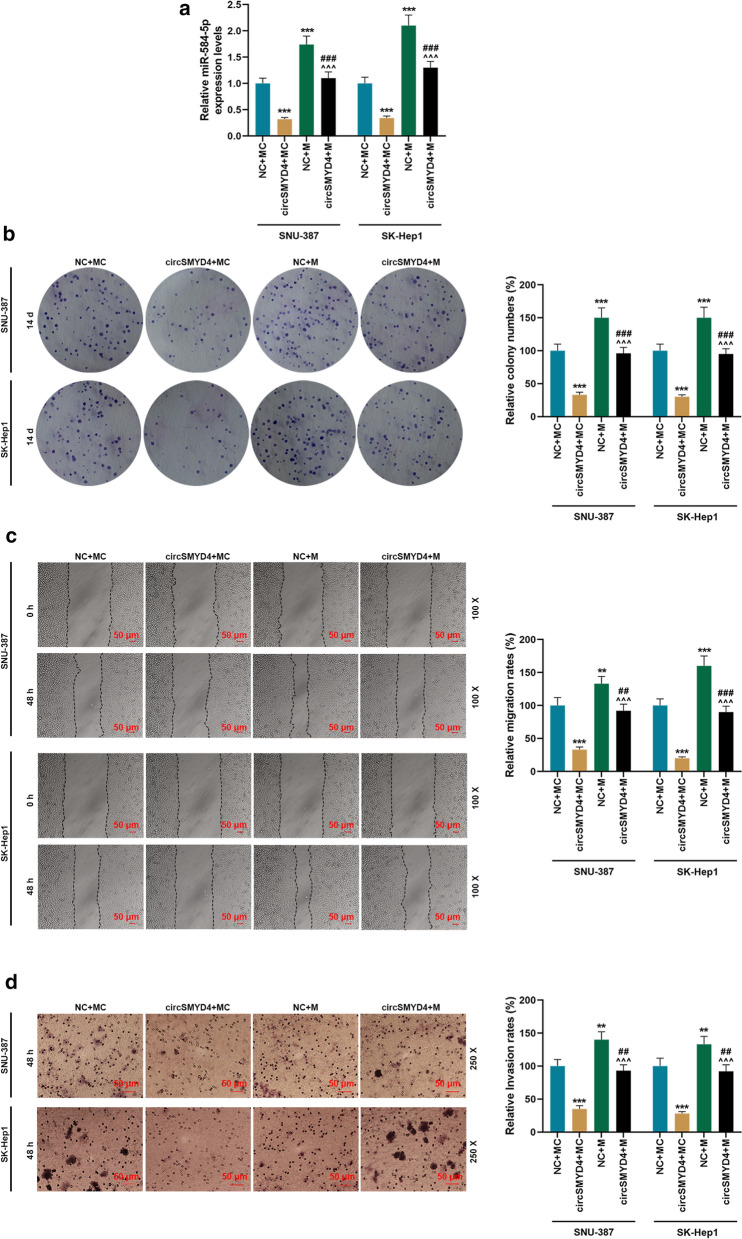
Overexpression of miR-584-5p partially reversed the effects of circSMYD4 on cell proliferation and metastasis. **a** In SNU-387 or SK-Hep1 cells, the expression of miR-584-5p in the NC + MC, circSMYD4 + MC, NC + M and circSMYD4 + M groups was detected by RT-qPCR. U6 served as a control. **b** The effect of miR-584-5p on the proliferation of cells in each group was examined using clone formation assay. **c** The effect of miR-584-5p on the migration ability of cells in each group was examined through wound healing experiments. Magnification × 100, Scale = 50 μm. **d** The effect of miR-584-5p on the invasion ability of cells in each group was examined by Transwell. Magnification × 250, Scale = 50 μm. Both biological and technical replicates were examined three times. ^**^p < 0.01, ^***^p < 0.001 vs. NC + MC; ^*^^^*^*p* < 0.001 vs. circSMYD4 + MC; ^*##*^*p* < 0.01, ^*###*^*p* < 0.001 vs. NC + M. RT-qPCR: reverse transcription real time quantitative polymerase chain reaction

**Fig. 6:**
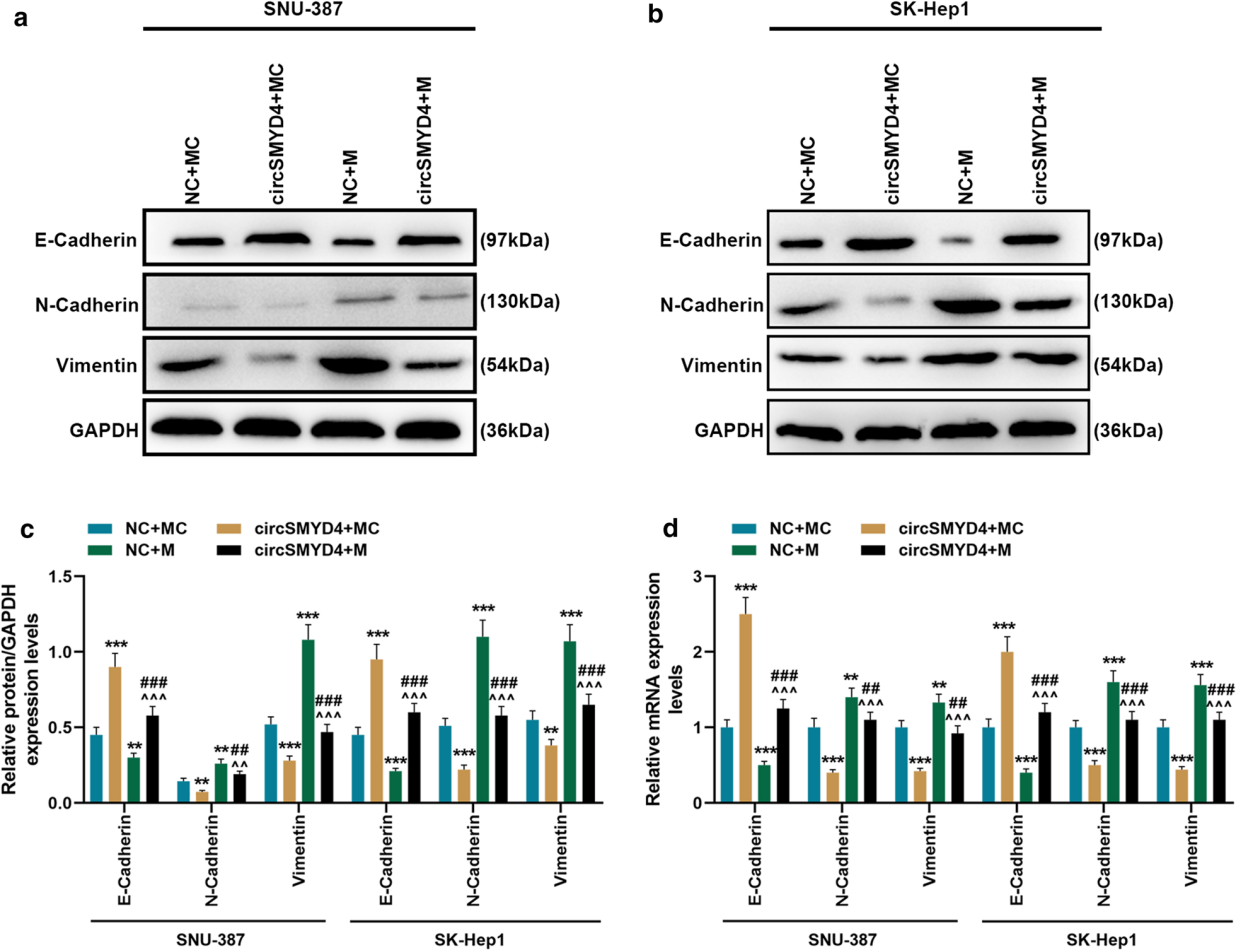
Overexpression of miR-584-5p partially reversed the effect of circSMYD4 on EMT-related genes. **a**–**c** Western blot was used to detect the expressions of EMT-related proteins in SNU-387 or SK-Hep1 cells in the NC + MC, circSMYD4 + MC, NC + M and circSMYD4 + M groups. **d** The mRNA expressions of EMT-related genes in each group of cells were analyzed by RT-qPCR. Both biological and technical replicates were examined three times. GAPDH was used as a control. The experiment was repeated for 3 times. ^**^p < 0.01, ^***^p < 0.001 vs. NC + MC; ^*^^*^*p* < 0.01, ^*^^^*^*p* < 0.001 vs. circSMYD4 + MC; ^*##*^*p* < 0.01, ^*###*^*p* < 0.001 vs. NC + M. RT-qPCR: reverse transcription real time quantitative polymerase chain reaction

The miR-584-5p mimic and circSMYD4 overexpression plasmids alone or in combination into SNU-387 and SK-Hep1 cells for further investigation (Fig. 5a). Compared with the NC + MC group, the number of cell clones decreased significantly in the circSMYD4 + MC group and increased significantly in the NC + M group, while the number of cell clones in the circSMYD4 + M group was significantly higher than that in the circSMYD4 + MC group (*p* < 0.001, Fig. 5b). The results of wound healing assay and Transwell showed that miR-584-5p mimic could partially reverse the effects of circSMYD4 on cell migration and invasion (*p* < 0.001, Fig. 5c, d). In addition, in SNU-387 and SK-Hep1 cells, circSMYD4 inhibited the expressions of N-Cadherin and Vimentin and promoted the expression of E-Cadherin (*p* < 0.01, Fig. 6a–d); while miR-584-5p mimic significantly reversed the regulatory effect of circSMYD4 on these EMT-related genes (*p* < 0.01, Fig. 6a–d).

### *CircSMYD4 targeted miR-584-5p to regulate tumor formation and apoptosis-related genes *in vivo

**Fig. 7:**
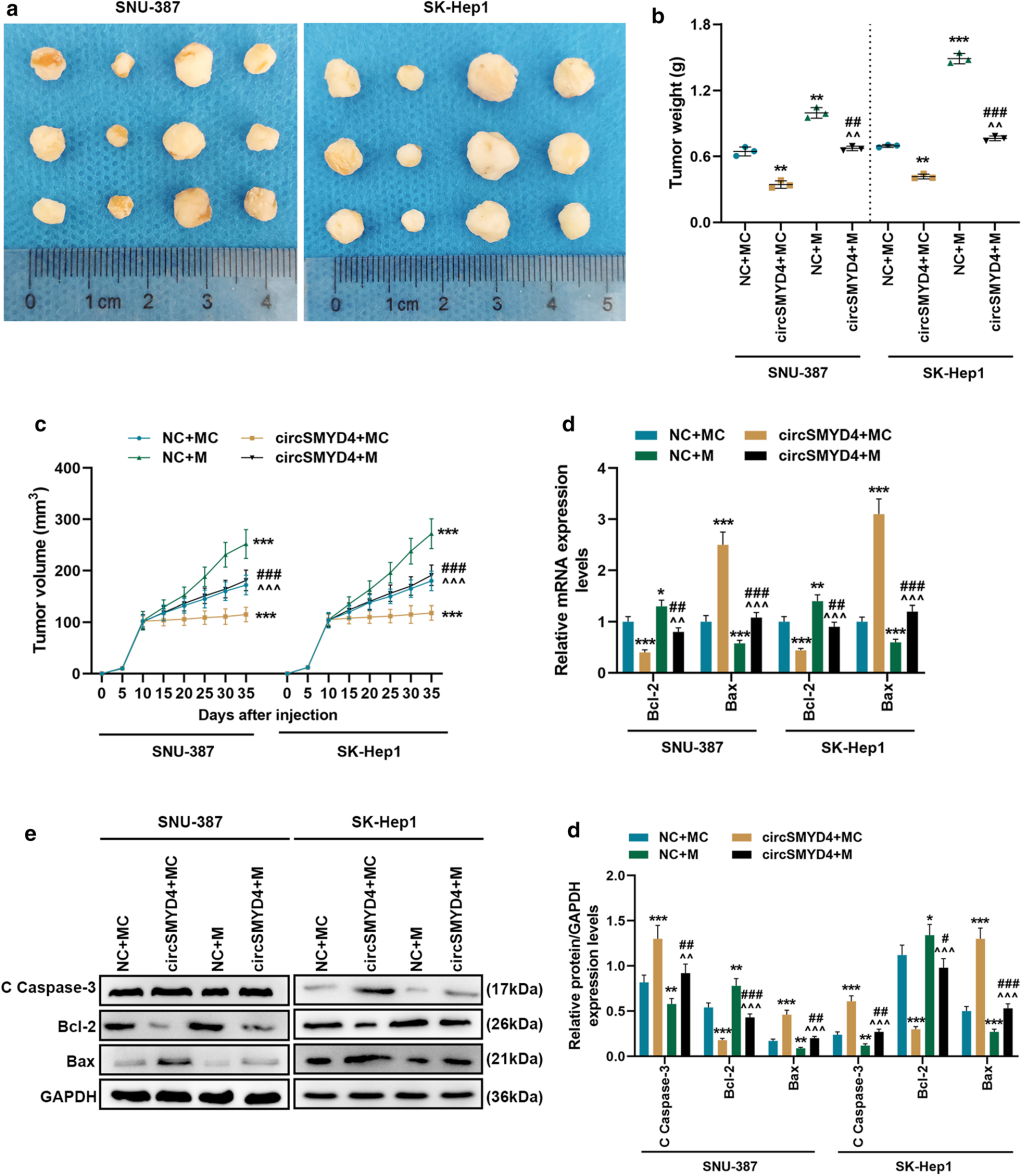
Effects of miR-584-5p and circSMYD4 on tumor formation and apoptosis-related genes in vivo. **a**–**c** Nude tumor formation experiments were conducted to examine the effects of miR-584-5p and circSMYD4 on tumor formation in vivo. **d** In SNU-387 or SK-Hep1 cells, the mRNA expressions of Bcl-2 and Bax in the NC + MC, circSMYD4 + MC, NC + M, and circSMYD4 + M groups were analyzed by RT-qPCR. **e**, **f** Western blot was performed to detect the expressions of cleaved Caspase-3, Bcl-2 and Bax proteins in SNU-387 or SK-Hep1 cells in each group. Both biological and technical replicates were examined three times. GAPDH was used as a control. Choose the three most representative data from the 8. The experiment was repeated for 3 times. ^**^p < 0.01, ^***^p < 0.001 vs. NC + MC; ^*^^*^*p* < 0.01, ^*^^^*^*p* < 0.001 vs. circSMYD4 + MC; ^*##*^*p* < 0.01, ^*###*^*p* < 0.001 vs. NC + M. RT-qPCR: reverse transcription real time quantitative polymerase chain reaction

In vitro experiments,compared with the control group, up-regulation of circSMYD4 substantially decreased the tumor weight and volume of nude mice with HCC, and miR-584-5p mimic could partially reverse the inhibitory effect of circSMYD4 overexpression on tumor growth (*p* < 0.01, Fig. 7a–c). In addition, circSMYD4 inhibited the expression of Bcl-2 and promoted the expressions of cleaved Caspase-3 and Bax, while miR-584-5p mimic had the opposite effect; and the regulatory effect of circSMYD4 on apoptosis-related genes was weakened in the circSMYD4 + M group (*p* < 0.05, Fig. 7d–f).

**Fig. 8:**
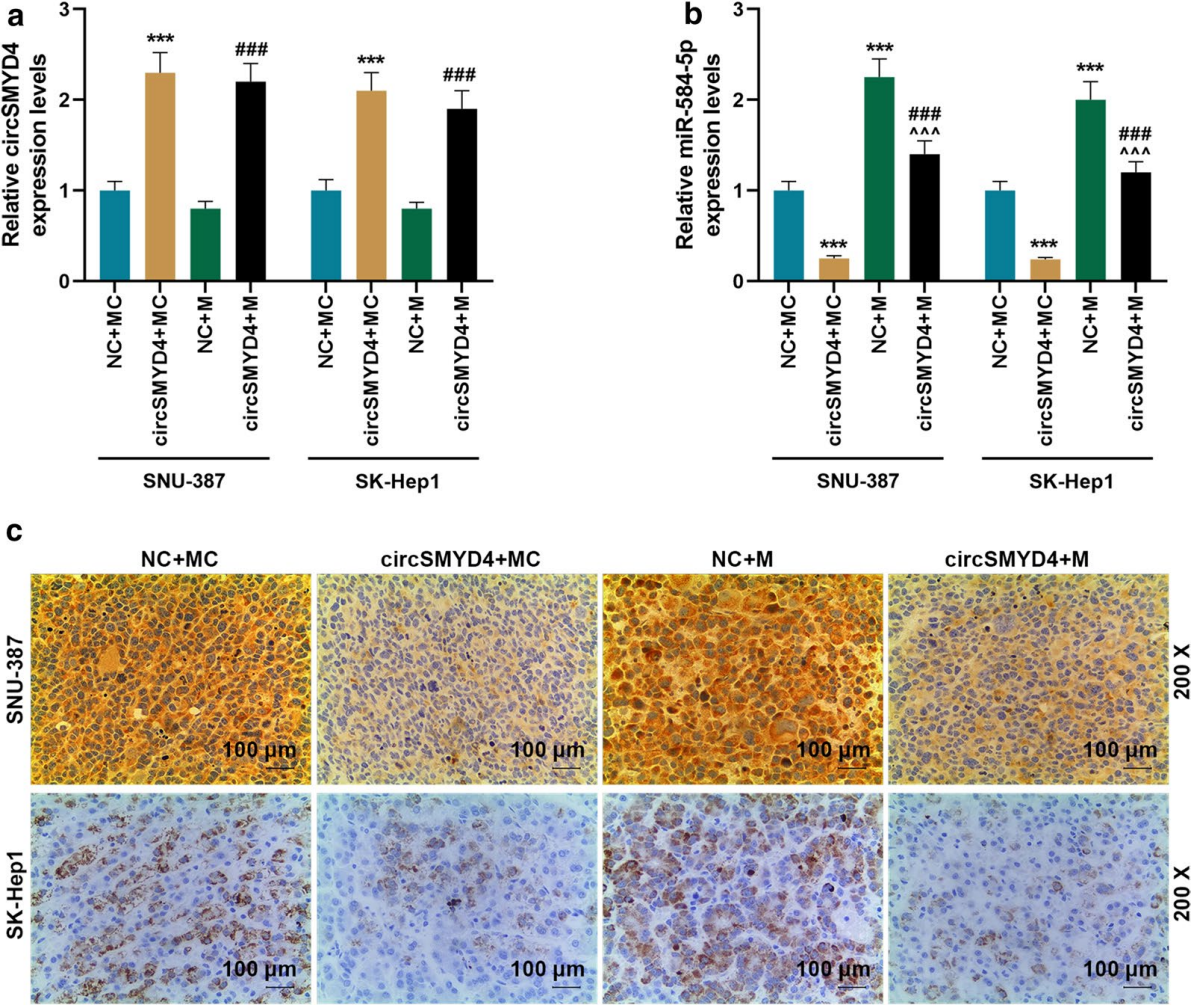
CircSMYD4 regulated the expression of alpha-fetoprotein (AFP) in vivo by targeting miR-584-5p. **a** MRNA expression of circSMYD4 in SNU-387 or SK-Hep1 cells in the NC + MC, circSMYD4 + MC, NC + M, and circSMYD4 + M groups were detected by RT-qPCR. GAPDH was used as a control. **b** The expression of miR-584-5p in each group was detected by RT-qPCR. U6 was used as a control. **c** Immunohistochemistry was used to detect the expression of AFP in each group. Magnification × 200, Scale = 100 μm. Both biological and technical replicates were examined three times. ^***^p < 0.001 vs. NC + MC; ^*^^^*^*p* < 0.001 vs. circSMYD4 + MC; ^*###*^*p* < 0.001 vs. NC + M. RT-qPCR: reverse transcription real time quantitative polymerase chain reaction

Next, we further detected the expressions of circSMYD4 and miR-584-5p in vivo, and found that compared with the NC + MC group, the expression of circSMYD4 was increased in the circSMYD4 + MC group, but that in the NC + M group remained unchanged (*p* < 0.001, Fig. 8a). Meanwhile, the expression of miR-584-5p decreased in the circSMYD4 + MC group and increased in the NC + M group, and was significantly higher in the circSMYD4 + M group than in the circSMYD4 + MC group, indicating that circSMYD4 negatively regulated miR-584-5p (*p* < 0.001, Fig. 8b). Finally, immunohistochemical results showed that compared with the control group, the number of AFP-positive cells in tumor tissues decreased in the circSMYD4 + MC group and increased in the NC + M group, and were significantly bigger in the circSMYD4 + M group than in the circSMYD4 + MC group (200 × magnification, Scale = 100 μm, Fig. 8c). These data suggested that up-regulation of circSMYD4 inhibited AFP expression in vivo by targeting miR-584-5p.

## Discussion

The occurrence of HCC is a complex process involving multi-factor interaction [22]. At present, most scholars believe that HCC is caused by genetic and epigenetic changes of multiple genes and the internal factors [23]. With the deepening of study on circRNA, there is more and more evidence that circRNA can be used as a post-transcriptional regulator to participate in the formation, metastasis and recurrence of HCC [12, 15]. CircSMYD4 is a recently reported gene that is significantly differentially expressed in HCC [16], but its molecular mechanism has not been reported; therefore, we explored the function and mechanism of circSMYD4 in HCC.

Interestingly, we found that the expression of circSMYD4 was significantly reduced in HCC tissues and cells, which is consistent with the findings of previous studies. CircSMYD4 is a circRNA transcribed from SMYD4, a member of the histone methyltransferase SMYD family [24, 25]. CircSMYD4 mainly regulates its downstream target genes and key tumor signaling pathways through histone or non-histone methylation modification, and participates in the entire process of tumorigenesis [26]. SMYD4 acts as a tumor suppressor gene in breast cancer cells and directly inhibits the gene expression of platelet-derived growth factor receptor A polypeptide (Pdgfr-A), thereby inhibiting tumor growth [27]. Studies have shown that in some cases, the abundance of circRNA can be more than 10 times that of related linear mRNA [28]. Our research showed that the closed-loop structure of circSMYD4 exhibits different characteristics from linear SMYD4. CircSMYD4 is a highly conserved circRNA with stable existence in cells and is not easily degraded by RNase R, which suggests that circSMYD4 is a promising biomarker and therapeutic target, and may be involved in regulating the biological functions of HCC cells.

Currently, a large number of studies have reported that circRNA plays a key role in the malignant phenotype of HCC cells [10]. In vitro experiments have shown that overexpression of circRNAs such as circ_0067934 and has_circ_0005075 can generally promote the proliferation and migration ability of HCC cells and inhibit their apoptosis [14, 29]. However, different circRNAs play different roles in HCC. Some scholars have found that overexpression of circADAMTS14 can inhibit the above malignant phenotype [30]. In this study, we found that circSMYD4 inhibited viability, clone formation, migration, and invasion, and promoted apoptosis by up-regulating the expression of circSMYD4, and further revealed that circSMYD4 acts as a powerful tumor suppressor gene in the occurrence of HCC.

It has been reported that exonic circRNAs, which mainly exist in the cytoplasm, account for the great majority of circRNAs and may function as miRNA sponges [31]. For example, Zeng et al*.* demonstrated that circHIPK3 acts as a sponge for “adsorb” miR-7 and regulates the expression of related target genes, thereby promoting the proliferation of colorectal cancer cells [18]. Our research also found that circSMYD4 is more abundant in the cytoplasm than in the nucleus, and that circSMYD4 contains many miRNA binding sites, suggesting that circSMYD4 may effectively bind to miRNAs, become miRNA “sponges”, and function as a competing endogenous RNA (ceRNA). CircSMYD4 may compete with miRNAs through its own miRNA response elements, regulate the expression levels of target genes, and ultimately affect the biological functions of HCC cells. Bioinformatics prediction, luciferase analysis, and Spearman correlation analysis showed that circSMYD4 negatively regulates miR-584-5p in HCC cells. MiR-584-5p has been found to be abnormally expressed in a variety of tumors and is associated with tumor cell proliferation and metastasis [32, 33]. We further studied the roles of miR-584-5p and circSMYD4 through in vitro experiments and found that overexpression of miR-584-5p partially reversed the promoting effect of circSMYD4 on cell proliferation and metastasis. Multiple studies have confirmed that EMT is an important step in tumor invasion and metastasis, and it is considered as a marker for tumor metastasis [34, 35]. We also detected EMT-related proteins and further demonstrated that circSMYD4 inhibits the metastasis of HCC cells by targeting miR-584-5p. Next, we studied the effects of miR-584-5p and circSMYD4 in vivo. As expected, it was found that overexpression of circSMYD4 targeted the inhibition of miR-584-5p expression, reduced AFP levels, and significantly slowed tumor growth in HCC. Therefore, we speculated that the mechanism of circSMYD4 in HCC may be that circSMYD4 can down-regulate the expression of miR-584-5p and then regulate multiple signaling pathways such as metastasis and apoptosis, thereby preventing the development of HCC.

In conclusion, this study found that circSMYD4 is low expressed in HCC cells and tissues, and as preliminary demonstrated, circSMYD4 regulates the proliferation, migration, and apoptosis of HCC cells by sponging miR-584-5p and plays a tumor suppressive role in HCC.

## Data Availability

The analysed data sets generated during the study are available from the corresponding author on reasonable request.
